# High calcium and strontium uptake by the green microalga *Tetraselmis chui* is related to micropearl formation and cell growth

**DOI:** 10.1111/1758-2229.13124

**Published:** 2022-09-23

**Authors:** Inés Segovia‐Campos, Montserrat Filella, Karl Perron, Daniel Ariztegui

**Affiliations:** ^1^ Department of Earth Sciences University of Geneva Geneva Switzerland; ^2^ Department F.‐A. Forel University of Geneva Geneva Switzerland; ^3^ Microbiology Unit University of Geneva Geneva Switzerland

## Abstract

Strontium‐rich micropearls (intracellular inclusions of amorphous calcium carbonate) have been observed in several species of green microalgae within the class Chlorodendrophyceae, suggesting the potential use of these organisms for ^90^Sr bioremediation purposes. However, very little is known about the micropearl formation process and the Ca and Sr uptake dynamics of these microalgae. To better understand this phenomenon, we investigated, through laboratory cultures, the behaviour of two species within the class Chorodendrophyceae: *Tetraselmis chui*, forming micropearls, and *T. marina*, not forming micropearls. We show that *T. chui* growth and micropearl formation requires available Ca in the culture medium, and that the addition of dissolved Sr can partially replace the function of Ca in cells. On the other hand, *T. marina* can grow without added Ca and Sr, probably due to its inability to form micropearls. *T. chui* cells show a high Ca and Sr uptake, significantly decreasing the concentration of both elements in the culture medium. Strontium is incorporated in micropearls in a short period of time, suggesting that micropearl formation is, most likely, a fast process that only takes a few hours. In addition, we show that micropearls equally distribute between daughter cells during cell division.

## INTRODUCTION

Chlorodendrophyceae are a class of green microalgae widespread in aquatic environments including seawater, freshwater, and brackish water (Norris et al., 1980). This class is composed of two genera: *Scherffelia* and *Tetraselmis* (Guiry & Guiry, 2021), the latter commonly used as a feed in aquaculture to support the growth of bivalve molluscs (Meseck et al., 2005). Over the last decade, the combination of electron microscopy with adapted sample preparation methods has revealed the formation of intracellular inclusions of amorphous calcium carbonate (ACC) in most of the species within this class (Martignier et al., 2018, 2020; Segovia‐Campos et al., 2021). These mineral inclusions are called micropearls and their distribution, shape, and number per cell vary according to the chloroplast morphology of the species and their habitat (Martignier et al., 2020). The differences among species coincide with the 5.8/ITS2‐ and *rbcL*‐based phylogenetic trees, making micropearls a valid criterion to distinguish the different clades within the class Chlorodendrophyceae.

ACC is the most unstable calcium carbonate polymorph, being relatively soluble in its pure state (Addadi et al., 2003). Micropearls ACC can contain a high percentage of Sr compared to the chemical composition of the extracellular medium (Martignier et al., 2018). This finding suggests that Chlorodendrophyceae have the ability to selectively concentrate Sr (Martignier et al., 2018; Segovia‐Campos et al., 2021), being of potential interest to develop new strategies to efficiently remediate ^90^Sr, which is a major radioactive pollutant released into the environment in the event of a nuclear accident (Vakulovsky et al., 1994).

In the past years, similar mineral inclusions have also been reported in very diverse microorganisms, including both prokaryotes and unicellular eukaryotes (protists) (Segovia‐Campos et al., 2021). For instance, some cyanobacteria such as *Gloeomargarita lithophora* and *Candidatus* Synecococcus calcipolaris form ACC inclusions in which Ba and Sr are preferentially accumulated over Ca (Cam et al., 2016). Non‐identified protists from Lake Geneva (France, Switzerland) also present ACC inclusions highly enriched in Sr and Ba (Martignier et al., 2017). However, high concentrations of Ba have never been detected in Chlorodendrophyceae micropearls.

The discovery of these intracellular Sr and/or Ba‐enriched ACC inclusions in both prokaryotic and eukaryotic microorganisms is of great interest as it shows that the intracellular biomineralization of calcium carbonate is more widespread than previously thought. Indeed, the biogenic formation of calcium carbonate in microorganisms has commonly been considered an extracellular process, the description of intracellular calcium carbonates being rare in the past. Only a few microorganisms were known to form intracellular calcium carbonates: coccolithophores (Brownlee et al., 2020), the giant sulfur bacterium *Achromatium* (Gray & Head, 2014; Head et al., 2000), and some aquatic ciliates (Fauré‐Fremiet & Gauchery, 1957).

Several studies have attempted to identify the physiological function of the intracellular inclusions of ACC in microorganisms and the molecular mechanisms behind their formation and stabilization. It has been suggested that the ACC mineral inclusions in cyanobacteria could (i) have a role in the regulation of intracellular pH and alkalinity (Benzerara et al., 2014; Couradeau et al., 2012; De Wever et al., 2019), (ii) act as ballast by increasing cell density to adapt the position of these microorganisms in the water column (Couradeau et al., 2012; De Wever et al., 2019), (iii) constitute carbon reserves (De Wever et al., 2019), and/or (iv) be involved in cell division (Benzerara et al., 2014). In the green microalgae within the class Chlorodendrophyceae, micropearls have been suggested to constitute Ca deposits that could be linked to the formation of the flagella and theca, as well as to the motility and buoyancy of the cells (Martignier et al., 2020). However, there is no evidence supporting these hypotheses and further research is needed to determine the real function of micropearls.

Some studies have investigated the possible impact of microorganisms forming intracellular ACC inclusions on the geochemical cycles of alkaline earth metals. The cyanobacterium *G. lithophora* has been shown to significantly impact the geochemical cycles of Ba and Sr in laboratory experiments in which batch cultures were supplemented with a continuous supply of alkaline earth metals by microbialite dissolution (Blondeau, Benzerara, et al., 2018). In the case of Chlorodendrophyceae microalgae, a few studies previous to the discovery of micropearls already pointed out high Sr concentrations in the cell content of some *Tetraselmis* species (Mei et al., 2006; Ulloa et al., 2012). It has been hypothesized that the formation of micropearls in Chlorodendrophyceae requires high Ca and Sr uptake by these organisms, which may alter the chemical composition of the extracellular medium (Martignier et al., 2017). However, again, this has never been experimentally proven.

In this study, we explore for the first time the micropearl formation process in Chlorodendrophyceae under controlled conditions. For this purpose, we compared the behaviour under different culture conditions of two seawater species within the class Chlorodendrophyceae: *Tetraselmis chui*, forming micropearls, and *Tetraselmis marina*, not forming micropearls. We follow (i) the effect of dissolved Ca and Sr concentrations on cell growth and micropearl formation, (ii) the location and distribution of micropearls during cell division, and (iii) the Ca and Sr uptake kinetics by Chlorodendrophyceae and their impact on the chemical composition of the culture medium. Micropearl formation was studied using scanning electron microscopy (SEM) in conjunction with energy‐dispersive x‐ray spectroscopy (EDXS), while cation uptake was mainly followed by simultaneous measurement of their concentrations in cells and culture media using inductively coupled plasma mass spectrometer (ICP‐MS).

## EXPERIMENTAL PROCEDURES

### Origin, selection, and characteristics of the studied strains

*Tetraselmis chui* (8‐6) and *Tetraselmis marina* (202.80) strains were both obtained from the Culture Collection of Algae at the University of Göttingen (SAG) (Table S2). *Tetraselmis chui* was chosen to study micropearl formation as its growth is relatively fast and its micropearl distribution pattern the most commonly observed within the class Chlorodendrophyceae. *Tetraselmis marina* was selected for this study as it is one of the few Chlorodendrophyceae species that do not form micropearls, allowing direct comparison between a micropearl‐forming and a non‐micropearl‐forming species within the same genus.

### Culture conditions

ASP‐H medium (Littler et al., 1973) modified by McFadden and Melkonian (1986) was prepared without added Ca at pH 8.3 and autoclaved (Table S3). Filtered SrCl_2_·6H_2_O and CaCl_2_·2H_2_O stock solutions were subsequently added to obtain culture media with different Ca and Sr concentrations. To test Ca and Sr uptake capacities of *T. chui* and *T. marina*, as well as their behaviour under different culture conditions, both strains were separately inoculated in 400 ml of the different culture media at a cell density of ~10^4^ cell ml^−1^. The cultures were set in triplicates using 2 L capacity Erlenmeyer flasks and placed in an incubator (Multitron Standard‐ Infors HT) during 360 h (15 days) at 20°C, 110 rpm shaking, and a 14:10 light–dark cycle with 400 lux light intensity. Non‐inoculated media were placed under the same growth conditions as controls. All pre‐cultures were grown in a culture medium with 2.5 mM Ca, which is the standard Ca concentration of ASP‐H modified medium. For some of the experiments presented in study, we also cultured the algae with lower and higher Ca concentrations (0, 0.1, 0.5, 4, and 16 mM) in order to investigate the optimal Ca concentrations for cell growth and micropearl formation. Depending on the experiment performed, the Sr concentrations added to the culture medium were 0, 0.1, and 0.5 mM. The concentration of 0.1 mM corresponds to the natural Sr concentration in seawater (Luther, 2016), while 0.5 mM Sr was the chosen concentration to investigate the effect of a higher (but environmentally plausible) Sr concentration on the algae behaviour.

### Sampling

Fifty‐two‐millilitres samples of the microalgae cultures were collected every 72 h. From this volume, 1 ml was used to measure the cell concentration of the cultures, 200 μl to prepare samples for scanning electron microscopy (SEM), and 50 ml for chemical analysis by inductively coupled plasma mass spectrometry (ICP‐MS). In order to follow the incorporation of Sr in micropearls using SEM, we also sampled 200 μl of the cultures enriched with 0.5 mM Sr after 1, 3, 5, 9, 12, and 24 h from the Sr addition.

### Cell concentration measurements

Cell concentration in liquid cultures was estimated by measuring their optical density at 680 nm, using a spectrophotometer (WPA, Biowave). For each species, a correlation between optical density and cell density was previously established after cell counting in a Neubauer chamber.

### SEM sample preparation, observation, and energy‐dispersive x‐ray spectroscopy (EDXS) analysis

Samples for SEM observation were prepared by filtering 200 μl of the liquid cultures with a 1–2 μm pore size polycarbonate membrane (Whatman® Nuclepore™). During filtration, a weak vacuum pressure was applied to avoid cell deformation and breakage. The filter was then placed in an Al stub with a double‐sided carbon tape and coated with a thin layer of Au (10 nm) by low vacuum pressure sputtering.

Sample observation and imaging were performed with a JEOL JSM 7001F Scanning Electron Microscope equipped with an EDXS detector (Model EX‐94300S4L1Q, JEOL) for semi‐quantitative analysis. Samples were observed and imaged using the backscattered electron detection mode. EDXS measurements were acquired with a beam current of 7 nA, 15 kV accelerating voltage and an acquisition time of 30 s. Semi‐quantitative data were obtained by applying the ZAF correction. Results are given as mole percent. Micropearls Sr/Ca mol% ratios measurements in *T. chui* samples were performed in at least 25 micropearls (randomly selected). The obtained values were then used to calculate the average Sr/Ca mol% ratios and the standard deviation of each dataset.

Micropearls length measurements were performed using the software ImageJ (Schneider et al., 2012). We compared the micropearls length of *T. chui* cells grown with 2.5 mM Ca and different Sr concentrations: 0 mM (*n* = 105), 0.1 mM (*n* = 44), and 0.5 mM (*n* = 46) by performing a Tukey HSD statistical test using the Vassar Statistics web server (Lowry, 2015). We previously applied the Shapiro*–*Wilk statistical test to confirm the normal distribution of the samples using the software GraphPad Prism 8 for Windows (GraphPad Software, San Diego, California, USA).

### Calcium and strontium concentration measurements

For chemical analysis, 50 ml samples of *T. chui* and *T. marina* cultures were centrifuged in 50 ml sterile polypropylene tubes (Falcon®) at 4000 rpm during 5 min (Sorvall Legend X1R, Thermo Scientific). Five millilitres of the supernatants (corresponding to the culture media) were then stored in 15 ml sterile polypropylene tubes (Falcon®) at −80°C for further analyses. The rest was discarded. Algae pellets were transferred to 1.5 ml Eppendorf tubes® and rinsed three times with 1 ml of a solution containing Milli‐Q® water, 0.1 M NaCl, and 2 mM HEPES (adjusted at pH 8.3). After each rinse, the tubes were centrifuged for 1 min at 4000 rpm (Eppendorf minispin pro) and supernatants discarded. Washed algae pellets were immediately frozen in liquid nitrogen and freeze‐dried (Alpha 2‐4, Christ) for 24 h.

Ten milligrams of each sample of lyophilized algal cells were accurately weighted using a microbalance (XPR 26, Mettler‐Toledo) and placed in 4 ml borosilicate tubes (Wheaton®) with 256 μl 65%–67% HNO_3_ (Normaton) and 64 μl 30% H_2_O_2_ (Supelco) to carry out microwave‐assisted acidic digestions (Multiwave Pro Solv, Anton Paar). Four samples of Certified Reference Material (CRM) No. 3 *Chlorella* (National Institute for Environmental Studies, Japan) were prepared following the same procedure to test the effectiveness of the digestion. Rotor 64MG5 was loaded with samples, CRM, and blanks (65%–67% HNO_3_/30% H_2_O_2_ = 4:1). The digestion programme applied was (i) 15 min ramp to reach 150 W, (ii) hold 15 min at 150 W, (iii) 20 min ramp to reach 300 W, (iv) hold 30 min at 300 W. The IR‐limit was set at 140°C. Digested algae samples and blanks were first diluted 50 times in Milli‐Q® water, and then diluted a second time with 2% HNO_3_ to obtain a final dilution factor of 1500 in a 2% HNO_3_ matrix. To obtain good Ca and Sr detection levels, CRM digested solutions were diluted only 50 times in Milli‐Q® water. Culture media samples were thawed at room temperature and diluted 80 times in 2% HNO_3_. The CRM *Sea Water* (High‐purity standards) was diluted following the same process. Calibration solutions were prepared with Ca and Sr reference solutions that were diluted in 2% HNO_3_. Quality controls (QCs) used during the analyses of the digested algae samples were prepared using a 2% HNO_3_ matrix spiked with different volumes of Ca and Sr standard solutions. QC solutions used for the analyses of the culture media were prepared with a matrix containing non‐inoculated ASP‐H modified medium (without Ca and Sr) that was diluted 80 times in 2% HNO_3_ and was spiked with different concentrations of Ca and Sr. Before running the analyses, all the solutions were filtered with 0.5 μm pore size polytetrafluorethylene (PTFE) filters (Puradisc). Isotopes ^43^Ca, ^44^Ca, ^87^Sr, and ^88^Sr were measured in all the samples using ICP‐MS (7700x, Agilent Technologies) in No Gaz, Helium, and High Energy Helium modes. The instrument was coupled to an ASX‐500 Series autosampler (Agilent Technologies). A solution containing 50 μg L^−1^ of both Re and Rh was employed as an internal standard to detect and correct any plasma fluctuations. Calcium and Sr calibrations were performed separately to detect and correct any Sr interference in Ca measurements.

Analyses of CRM and QC samples provided satisfactory results: accuracies for both Ca and Sr concentrations ranged between 90% and 110% with a degree of precision (RSD) below 10%. Instrumental detection limits (3 × blank standard deviation) for Ca and Sr measurements were 2.9 and 0.06 μg L^−1^, respectively.

### Calculation of calcium and strontium uptake rates

Calcium and Sr uptake rates, *UR*, were calculated as (Cam et al., 2016):

$${\mathrm{UR}}_{j}=\frac{\frac{{\left[X\right]}_{i}-{\left[X\right]}_{j}}{{\bar{\mathrm{CD}}}_{\mathrm{ij}}}}{t_{j}-t_{i}}$$

where *t* is the time (in h), *i* and *j* two successive measurement times, [*X*] is the concentration of the element X (in fM) in the culture medium, $\bar{\mathrm{CD}}$ is the average cell density between times *i* and *j* (in cell L^−1^).

## RESULTS

### *Effect of the initial calcium and strontium concentration on* T. chui *and* T. marina *growth*

**FIGURE 1:**
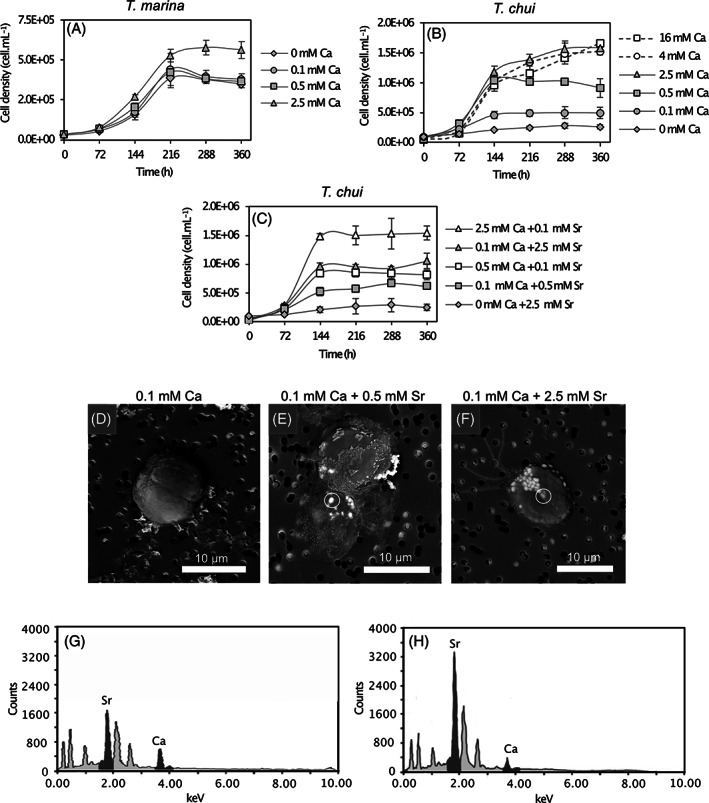
(A, B) Growth curves of (A) *T. marina* and (B) *T. chui* cultures in ASP‐H medium with different initial concentrations of dissolved Ca and without added Sr. (C) Growth curves of *T. chui* cultures in ASP‐H medium with different initial concentrations of dissolved Ca and Sr. Error bars represent standard deviations (*n* = 3). (D–F) SEM images of *T. chui* cells after 288 h of culture in ASP‐H medium with different initial concentrations of dissolved Ca and Sr. Micropearls are observed as bright and spherical to elongated bright bodies (≤1 μm) within the cells. Dark dots outside the cells correspond to the filter pores. (D) A dividing *T. chui* cell cultured with 0.1 mM Ca and without Sr. Micropearls are not observed. (E) *T. chui* cells cultured with 0.1 mM Ca and 0.5 mM Sr presenting a few micropearls. (F) A *T. chui* cell cultured with 0.1 mM Ca and 2.5 mM Sr. Many micropearls are observed in the anterior pole of the cell. (G) EDXS analysis of the micropearl encircled in image (E). (G) EDXS analysis of the micropearl encircled in image (F).

To test if low Ca concentrations in the culture medium have a higher impact on *T. chui* (micropearl‐producer) growth than on *T. marina* (non‐micropearl‐producer), we followed the growth of both species in ASP‐H medium with different initial Ca concentrations in the medium. The growth of *T. chui* was strongly limited by low Ca concentrations in the medium compared to *T. marina* (Figure 1A,B). Indeed, *T. chui* cells were barely dividing in a culture enriched with 0.1 mM Ca. However, the addition of 0.5 and 2.5 mM Sr to the media allowed *T. chui* cell growth (Figure 1C).

To understand whether Ca and Sr have a similar effect on *T. chui* growth, we also followed its development in ASP‐H medium with different initial concentrations of dissolved Ca and Sr. In *T. chui* cultures without added Ca, the addition of Sr was not enough to trigger cell division (Figure 1C). Furthermore, *T. chui* cultures with 2.5 and 0.1 mM added Ca and Sr, respectively, showed a higher cell production than cultures in which these concentrations were switched (0.1 mM Ca and 2.5 mM Sr). Similar observations were made when the cell growth of *T. chui* cultures enriched with 0.5 mM Ca and 0.1 mM Sr was compared to that of cultures in which these concentrations were reversed (Figure 1C).

### Micropearls presence and composition

To investigate whether the availability of Ca and Sr in the culture medium can affect the micropearl formation process, we followed the formation of micropearls in *T. chui* cells cultured with different concentrations of both elements. SEM observation of *T. chui* cells cultured in ASP‐H medium enriched with 0.1 mM Ca showed that micropearls were absent under these culture conditions (Figure 1D). However, the addition of 0.5 and 2.5 mM Sr resulted in the formation of Sr‐rich micropearls (Figure 1E–H). A concentration of 0.5 mM dissolved Ca in *T. chui* cultures was also enough to trigger micropearl formation (Figure S1). *T. marina* cells were also observed under the same culture conditions and did not form micropearls in any case (Figure S2).

**FIGURE 2:**
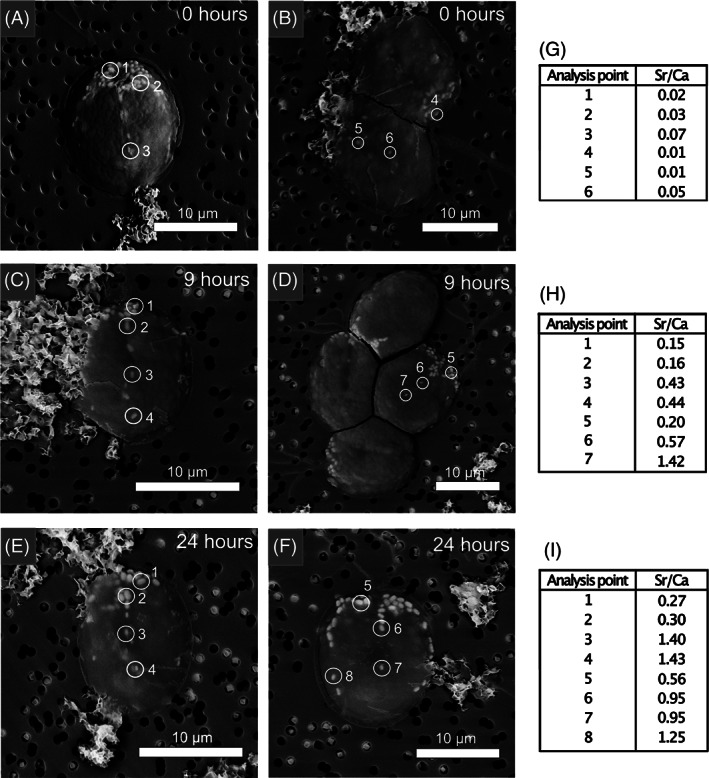
SEM images and EDXS analyses results of *T. chui* cells cultured in a medium enriched with 2.5 mM Ca, spiked with 0.5 mM Sr during the exponential growth stage of the culture. Images were obtained after: (A, B) 0, (C, D) 9, and (E, D) 24 h from the Sr addition. EDXS analysis areas are encircled. (G) Sr/Ca atomic ratios measured in images (A) and (B). (E) Sr/Ca atomic ratios measured in images (C) and (D). (F) Sr/Ca atomic ratios measured in images (E) and (F).

**FIGURE 3:**
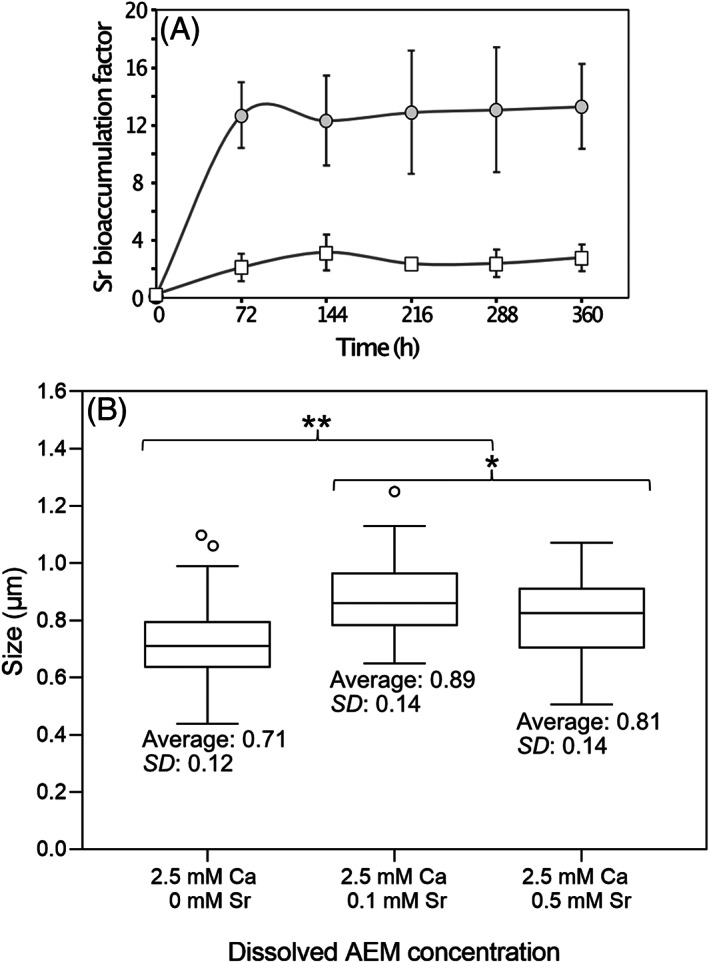
(A) Time evolution of Sr bioaccumulation factors measured in micropearls (*n* ≥ 25) in *T. chui* cultures enhanced with 2.5 mM Ca and 0.1 mM Sr (grey circles) and 2.5 mM Ca and 0.5 mM Sr (open squares). Strontium bioaccumulation factors were calculated by dividing the Sr/Ca mol% ratio of micropearls (EDXS measurements) by the Sr/Ca mol% ratio of the culture medium (ICP‐MS measurements). (B) Tukey boxplot summarizing micropearl length measured in *T. chui* cells obtained from 288‐h‐old cultures enriched with different Sr initial concentrations. Data set includes length values of 105 micropearls of cultures enhanced with 2.5 mM Ca, 44 micropearls of cultures enhanced with 2.5 mM Ca and 0.1 mM Sr, and 46 micropearls of cultures enhanced with 2.5 mM Ca and 0.5 mM Sr. The bold lines indicate the median values, the bottom and top of the boxes span the second and third quartile, and the whiskers span 1.5 times the extend of the boxes. Open circles are outliers. The average of the length values and standard deviations (SD) are indicated for each boxplot. The Tukey HSD statistical test shows a significant (**p < 0.01) difference between cultures without Sr and cultures enhanced with this element. In addition, a less significant difference (*p < 0.05) is shown between the cultures enriched with 0.1 and 0.5 mM Sr.

EDXS analyses showed that Sr/Ca mol% ratios were rapidly established in *T. chui* micropearls after cell inoculation in a culture medium containing dissolved Ca and Sr and did not vary after 72 h of growth (Figure S3). Indeed, *T. chui* cells grown in cultures enhanced with 2.5 mM Ca and spiked with 0.5 mM Sr during the exponential growth stage already contained Sr‐rich micropearls 9 h after the Sr addition, being the Sr accumulation higher in the smaller micropearls mainly located in the centre of the cells (Figure 2). Finally, *T. chui* cells cultured with 2.5 mM Ca and 0.1 mM Sr showed the highest Sr/Ca mol% ratios in micropearls compared to the element ratio in the culture medium. Under these culture conditions, the average Sr/Ca mol% ratio measured in micropearls from *T. chui* cultures older than 72 h was 12.4 (±1.2) times higher than the ratio measured in the culture medium. This enrichment factor remained constant over time since not significant variations were observed (Figure 3A). In *T. chui* cultures enriched with 2.5 mM Ca and 0.5 mM Sr, the micropearls Sr/Ca mol% ratio was, on average, 2.5 (±0.9) times higher than the measured Sr/Ca mol% ratio in the culture medium. This value also remained constant over time (Figure 3A). Micropearls Sr/Ca mol% ratios of *T. chui* cultures enhanced with 0.1 mM Ca and 2.5 mM Sr, as well as with 0.1 mM Ca and 0.5 mM Sr, presented lower enrichment values (Table S1).

**FIGURE 4:**
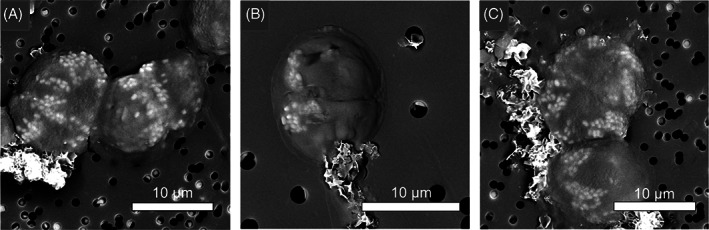
SEM images of *T. chui* cells showing an equal distribution of micropearls among daughter cells during cell division. (A) Micropearls in the cell located on the left side of the image are distributed in the central area of the cytoplasm of the two future daughter cells. The dividing cell located on the right side of the image shows micropearls along to the septation line. (B) Micropearls in the future daughter cells are located in opposites poles of the cell (since daughter cells are normally in an inverted position from each other during cell division; Gonzalez et al., 2015). (C) The future daughter cells located in the centre of the caption present micropearls in both poles of the cell.

*T. chui* cells produced micropearls 10% larger when Sr was added to the cultures compared to micropearls observed in *T. chui* cultures only enhanced with Ca (Figure 3B). SEM observation of dividing *T. chui* cells showed an equal distribution of micropearls between the daughter cells, each new cell receiving a similar number of micropearls (Figure 4). However, the spatial organization of micropearls was highly variable during cell division: micropearls could be scattered throughout the cytoplasm, distributed along the dividing line, or located in the cell poles.

### Kinetics of calcium and strontium uptake

**FIGURE 5:**
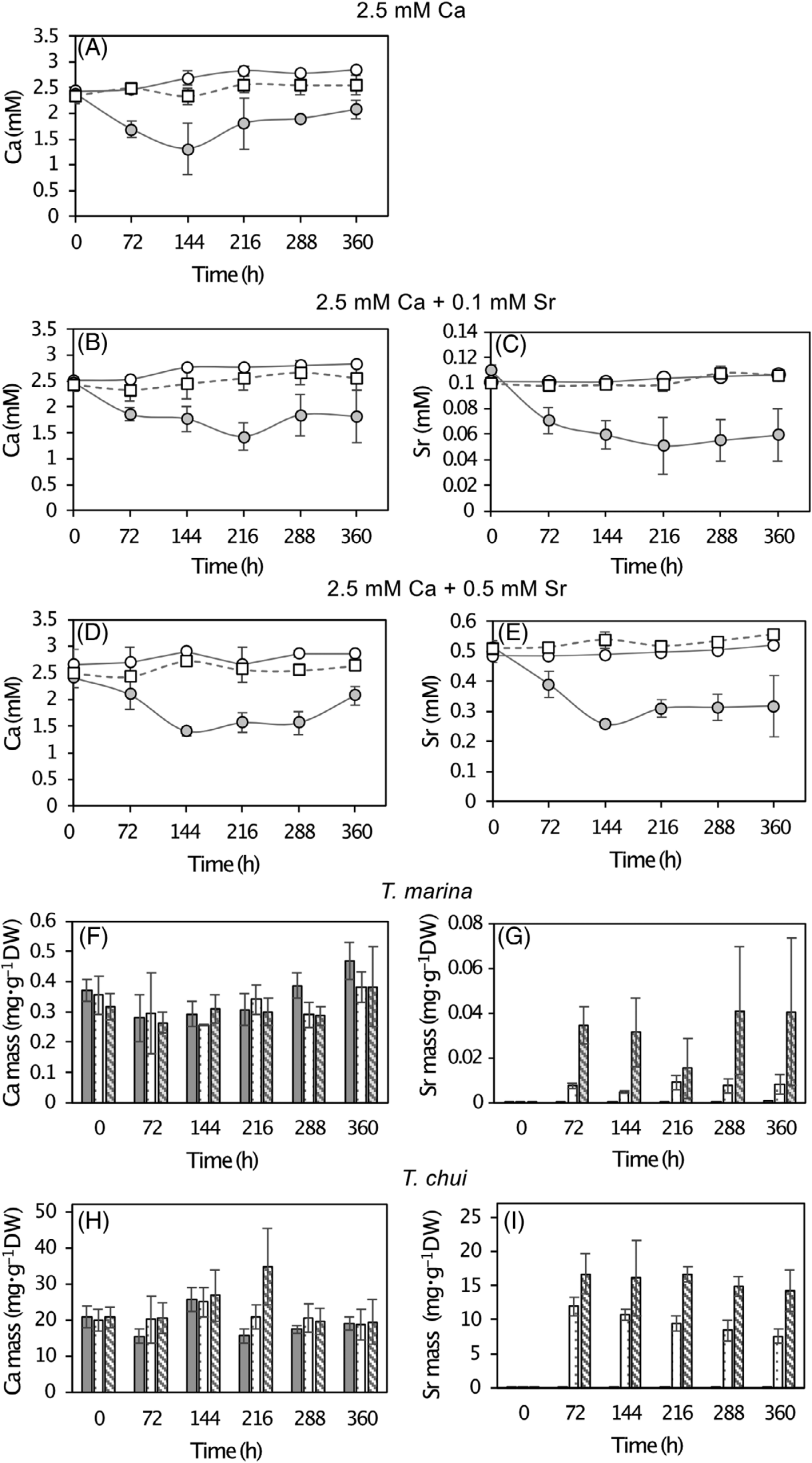
(A–E) Time evolution of dissolved Ca and Sr concentrations in *T. chui* (closed circles) and *T. marina* (open circles) cultures enhanced with different concentrations of both elements. (A) Dissolved Ca in cultures enriched with 2.5 mM Ca, (B) dissolved Ca and (C) dissolved Sr in cultures enhanced with 2.5 mM Ca and 0.1 mM Sr. (D) Dissolved Ca and (E) dissolved Sr in cultures enhanced with 2.5 mM Ca and 0.5 mM Sr. Controls (open squares) correspond to the dissolved Ca and Sr concentrations in non‐inoculated culture media. (F–I) Time evolution of the Ca and Sr total mass measured in *T. chui* and *T. marina* dried cells grown in cultures enhanced with: 2.5 mM Ca (grey bars), 2.5 mM Ca and 0.1 mM Sr (dotted bars), and 2.5 mM Ca and 0.5 mM Sr (striped bars). (F) Ca mass in *T. chui* cells. (G) Sr mass in *T. chui* cells. (G) Ca mass in *T. marina* cells. (I) Sr mass in *T. marina* cells. All pre‐cultures were already enriched with 2.5 mM Ca, which explains the high Ca content in cells at *t* = 0. Error bars represent standard deviations (*n* = 3).

We compared the Ca and Sr uptake capacities of *T. marina* and *T. chui* cells cultured with 2.5 mM Ca (standard Ca concentration of ASP‐H medium) and different Sr concentrations in order to test if high Ca and Sr absorption capacities are specific to micropearl forming species. By testing different Sr concentrations, we also investigated the best conditions for optimal absorption rates. In both species, cell growth was not altered by the different Sr additions in the culture media (Figure S4). In *T. marina* cultures and in non‐inoculated controls, the concentrations of dissolved Ca and Sr remained relatively constant over time (Figure 5A–E). However, the concentrations of dissolved Ca and Sr in *T. chui* cultures significantly decreased after 72 h of growth, reaching half of their initial concentrations after 144–216 h (Figure 5A–E), followed by a slight increase of the concentration of both dissolved elements during the stationary growth stage.

*T. chui* cells showed maximum absorption rates of both Ca and Sr after 72 h of culture, corresponding to the exponential growth phase of the algal cells (Figure S5). In all cultures, Ca and Sr uptake rates decreased over the duration of the culture, reaching values close to 0 fmol cell^−1^ h^−1^ after 216 h. The highest Ca uptake rate was observed in *T. chui* cultures in which Sr was not added, reaching an absorption rate of 50.2 (±15.2) fmol cell^−1^ h^−1^. Maximum Sr absorption rates (8.9 ± 3.9 fmol cell^−1^ h^−1^) were observed in *T. chui* cultures enhanced with 0.5 mM Sr, which is the highest concentration tested in this experiment. However, Ca uptake rate of *T. chui* cells cultured with 0.5 mM dissolved Sr was the lowest observed during the first hours of growth (22.2 ± 8.2 fmol cell^−1^ h^−1^). Indeed, we observed that Ca uptake rate in *T. chui* cells slowed down as Sr was added to the cultures.

Analysis of the cell content showed that Ca cellular concentrations were already stable in both species at *t* = 0 since all pre‐cultures had been enhanced with 2.5 mM Ca (Figure 5F–I). Calcium concentrations in the cells within the same species were similar in the different cultures, not being affected by the addition of Sr. The Ca concentration measured in *T. chui* cells reached values above 20 mg g^−1^ (Figure 5H), while the Ca concentration in *T. marina* cells was, on average, 67 times lower (Figure 5F). The Sr cell content was already stable in both species after 72 h. The highest Sr concentration measured in *T. chui* cells was 16.7 mg g^−1^ (Figure 5I) versus 0.04 mg g^−1^ measured in *T. marina* cells (Figure 5G), both grown in cultures enhanced with 0.5 mM Sr.

## DISCUSSION

### *Calcium and strontium high uptake by* T. chui *cells is related to micropearl formation and cell growth*

A relationship exists between the availability of Ca and Sr in the medium, the formation of micropearls, and the cell growth of *T. chui*. At low concentrations of dissolved Ca (0.1 mM) and absence of Sr, micropearls are absent in *T. chui* cells and cell growth is strongly limited compared to that of *T. marina* cultures, where micropearls have never been detected regardless of culture conditions. Interestingly, a similar Ca‐dependence has been previously noticed in some cyanobacteria forming intracellular ACC inclusions (De Wever et al., 2019). On the other hand, the addition of Sr in *T. chui* cultures with low initial dissolved Ca concentrations triggers the formation of micropearls (extremely rich in Sr) and cell growth. It is well known that Ca and Sr have similar chemical properties, and several studies have shown that Sr can substitute Ca in many functional processes in diverse organisms (Kylin & Das, 1967; Miledi, 1966; Walker, 1953). However, the growth rate of *T. chui* cultures in which Ca has been primarily replaced by Sr is slower than that of cultures where Ca has not been replaced. In addition, when Ca is totally absent, the presence of Sr is not enough to trigger micropearl formation and cell growth. Therefore, we can deduce that: (i) *T. chui* (and probably all micropearl‐forming species within the class Chlorodendrophyceae) only grows in environments where Ca is available. (ii) Micropearl formation and cell growth are dependent processes. (iii) When Ca is available at low concentrations in the cultures, Sr can partially replace the Ca function by promoting micropearl formation and cell growth.

Nevertheless, the ability of micropearl‐forming Chlorodendrophyceae to selectively concentrate Sr over Ca, might suggest that Sr itself has a role in the cells. Interestingly, the maximum Sr concentration capacity of *T. chui* occurs in cultures with Sr concentrations similar to those of seawater (Figure 3A and Table S1). Several studies have shown that the lack of available Sr can affect CaCO_3_ biomineralization in some organisms. For instance, in the opisthobranch gastropod *Aplysia californica*, embryos cultured in artificial seawater without Sr show deformed shells and statocysts lacking the statolith (Bidwell et al., 1986). In addition, the absence of Sr also affects the development of statoliths in hatchling cephalopods as well as the formation of the cuttlefish bone, indicating a possible significant role of Sr in carbonate biomineralization (Hanlon et al., 1989). Although Sr does not seem to play an essential role in the biomineralization of micropearls in *T. chui* cells, micropearls are 10% smaller when Sr is absent in the culture media compared to micropearls of cultures enhanced with 0.1 and 0.5 mM Sr (Figure 3b). This size difference is probably directly related to the Sr covalent radius being ~10% larger than that of Ca.

### Micropearl formation process

Thien et al. (2017) estimated the formation and growth of micropearls to be a process lasting between 14 h and 72 days, depending on the ambient temperature and the alkaline earth metal concentrations in the micropearls. Here, we found that Sr is incorporated in the micropearls in about 9 h and that Ca and Sr are mainly absorbed during the exponential growth stage of the algal cells, indicating that the uptake of these elements and the formation of micropearls occurs shortly after cell division. Hence, micropearl formation rather occurs on a time scale of a few hours. We have also observed that micropearls are equally distributed among the daughter cells as previously shown in cyanobacteria forming ACC inclusions (Li et al., 2016). To maintain a certain number of micropearls per cell after cell division, it seems reasonable to assume that the new micropearls will form rapidly. Indeed, after the addition of 0.5 mM Sr in *T. chui* cultures during the exponential growth stage, small inclusions highly enriched in Sr (compared to those located in the anterior part of the cells) are observed in the central zone of the cells, probably due to their recent formation compared to the larger micropearls situated in the anterior pole that are possibly older (Figure 2). This observation suggests that micropearls begin to form first in the central part of the cells and that, as they grow, they are transported to the anterior part of the cells. It is not yet known how the inclusions move inside cells; however, clear changes in the distribution pattern of intracellular inclusions have been observed over time in other species such as *Tetraselmis contracta* (Martignier et al., 2020). In addition, the fact that micropearls are not randomly segregated among the daughter cells during cell division indicates that their location is actively controlled by the cell and can be relocated. In cyanobacteria forming intracellular ACC inclusions, it has been postulated that the spatial organization of the inclusions within the cells may involve cytoskeletal proteins (Benzerara et al., 2014; Li et al., 2016).

It is well known that ACC formation takes place in highly supersaturated solutions (Aizenberg et al., 1996; Loste et al., 2003; Weiner & Dove, 2003). For that to occur, ions are transported to a specific cell location against concentration gradients (from unsaturated to saturated environments) (Mann, 2001). In cyanobacteria and other prokaryotic organisms forming intracellular ACC inclusions, the formation of the mineral inclusions has been shown to occur in intracellular compartments where saturation levels are probably regulated by transport proteins that actively control Ca, Sr, Ba, and carbonates import (Blondeau, et al., 2018; Monteil et al., 2020). Most likely, similar structures envelope micropearls in Chlorodendrophyceae microalgae, although this has not been proven yet. If so, Ca entry in the intracellular compartments could, for instance, involve Ca^2+^/H^+^ antiporters similar to those believed to occur in the membranes of coccolith vesicles of coccolithophorid algae, where calcite scales (coccoliths) are formed (Brownlee et al., 2020). In cyanobacteria‐forming ACC inclusions, at least one gene coding for this type of antiporters has been identified (De Wever et al., 2019). Recently, a new gene family encoding calcyanin proteins has been identified in cyanobacteria forming intracellular ACC inclusions, being possibly involved in Ca homeostasis and ACC formation (Benzerara et al., 2022). However, the specific function of these proteins is still not clear. Regarding Sr, it has been shown that many plants incorporate this element following Ca uptake pathways since they are not able to discriminate between these two elements (Drouet & Herbauts, 2008; Jovanović et al., 2021; Watanabe et al., 2007). However, we do not know yet if the import pathways of Sr to the nucleation sites are the same as those of Ca, or if other transport systems presenting high affinities for Sr are involved. Nevertheless, the fact that the Sr/Ca ratio is higher in the micropearls than in the culture media containing low Sr concentrations suggests that the Sr transport to the nucleation site is a selective process. Although such Sr‐transport systems have never been described before, they must also exist in organisms such as the planktonic unicellular eukaryotes *Acantharia*, presenting a skeleton made of SrSO_4_ (Decelle & Not, 2015), or the brown alga *Cystoseira barbata*, able to specifically concentrate Sr over Ca (Tashmukhamedov et al., 1983). We also observed that the addition of dissolved Sr in *T. chui* cultures slowed down the Ca uptake rate of this organism during the first 72 h, suggesting a possible Ca and Sr competition for the binding site of transporter proteins that could be located in the cell membrane. Finally, carbonates could enter in the intracellular compartments as HCO_3_^−^ and further dissociate in CO_3_^2−^ and H^+^. The latter being subsequently exported by the Ca^2+^/H^+^ antiporter systems, allowing to maintain a high alkalinity and basic pH within the compartments, both critical conditions for carbonate precipitation (Cam et al., 2015). Results of ongoing genomic analyses will be essential to decipher the molecular processes involved in the transport of these elements to the biomineralization site.

### *Impact of* T. chui *on the chemical composition of the extracellular medium*

Previous studies have detected very strong Ca, Sr, and Ba uptake capacities in several cyanobacteria strains forming intracellular ACC inclusions (Blondeau, et al., 2018; Cam et al., 2016; De Wever et al., 2019). The maximal Ca and Sr uptake rates of the cyanobacterium *G. lithophora*, which has been suggested to significantly impact the geochemical cycles of alkaline earth metals, were estimated at 0.05 and 0.07 fmol h^−1^ cell^−1^, respectively (Cam et al., 2016). Here, the maximum Ca and Sr uptake rates of *T. chui* are estimated to be respectively 50.2 and 8.86 fmol h^−1^ cell^−1^. These values cannot be directly compared with those of *G. lithophora* because differences in cell size must be considered in order to make valid comparisons between organisms. The total Ca mass in *T. chui* cells could represent more than 2% of the total cell dry mass, which is a similar Ca accumulation capacity to that measured in *G. lithophora* and other cyanobacteria forming ACC inclusions such as *T. elongatus* or *Cyanothece* sp. (2%–4%) (Cam et al., 2016; De Wever et al., 2019). This result is consistent with previously published studies analysing the mineral composition of *T. chui* (Barat Baviera et al., 2013; Tibbetts et al., 2015). On the other hand, *T. marina* cells only accumulated an average of 0.03% of Ca in mass and, therefore, the concentration of dissolved Ca in the cultures of this species did not significantly vary over time. This large difference between *T. chui* and *T. marina* uptake capacities is most likely due to the absence of micropearls in *T. marina* cells, which means that most of the Ca measured in *T. chui* is stored in the micropearls. The total Sr mass in *T. chui* reached a maximum of 1.7% of the total cell dry mass, which is four times lower than that measured in *G. lithophora* cells (although it must be considered that the growing conditions were different in each case). Still, the Sr absorbing capacity of *T. chui* is greater than that of *T. marina* (0.001% of the total dry mass) and other microalgae species not known to form ACC intracellular inclusions (Table 1), suggesting that most of the Sr measured in *T. chui* cells is also accumulated in the micropearls. Our results are consistent with SEM‐EDXS analyses, showing high Sr concentrations in the micropearls of *T. chui*. These observations suggest a possible impact of Chlorodendrophyceae species forming micropearls on the geochemical cycles of Ca and Sr. However, further research is needed to determine the degree of influence of these microorganisms on both Ca and Sr global cycles, considering the dynamics and circulation of these elements, as well as the natural occurrence of Chlorodendrophyceae in aquatic environments. Finally, it is important to note that, after cell death in Ca and Sr unsaturated aquatic environments, micropearls are expected to rapidly dissolve because of their amorphous character (Cam et al., 2018; Riding, 2012; Segovia‐Campos et al., 2021). This could explain the slight increase of dissolved Ca and Sr during the last hours of growth in some *T. chui* cultures.

**TABLE 1:** Maximum Sr content measured in different microalgal species

Microalga	Dissolved Sr (mM) in the culture	Sr in cells (mg g^−1^ DW)	References
*Tetraselmis suecica*	0.1	7.3 (±0.8)	Ulloa et al. (2012)
*Tetraselmis chui*	0.1	11.92 (±1.39)	This study
	0.5	16.71 (±2.97)	This study
*Tetraselmis marina*	0.1	0.009 (±0.003)	This study
	0.5	0.04 (±0.02)	This study
*Scenedesmus sp*.	0.1	0.5	Fuller and Hardcastle (1967)
	0.5	2	Fuller and Hardcastle (1967)
*Sphaerocystis sp*.	0.1	3.5	Fuller and Hardcastle (1967)
	0.5	7	Fuller and Hardcastle (1967)
*Chlorella pyrenoidosa*	0.1	0.07	Knauss and Porter (1954)
	0.5	0.4	Knauss and Porter (1954)

## CONCLUSIONS

The present study evidences a high Ca and Sr uptake by *T. chui* cells that is related to micropearl formation and cell growth. This high uptake has not been observed in the non‐micropearl‐forming species *T. marina*, its growth not being strongly affected by low dissolved Ca and Sr concentrations in the cultures.

Available Ca is essential for both *T. chui* cell growth and micropearl formation. However, when this element is not available in sufficient concentrations, the addition of dissolved Sr to the cultures can partially replace Ca function by triggering Sr‐rich micropearl formation and restoring cell division.

Although Sr does not appear to be crucial for micropearl formation when Ca is available in *T. chui* cultures, Sr is specifically accumulated in micropearls in less than 9 h. Indeed, Sr/Ca mol% ratio in these mineral inclusions can be up to 10 times higher than in the culture media. The reason why *T. chui* cells accumulate Sr in micropearls is not clear yet and further research is needed.

During cell division, micropearls location in *T. chui* cells is strongly controlled, leading to their equal distribution between the daughter cells. The high Ca and Sr accumulation rates observed during the exponential phase of *T. chui* growth, as well as the rapid incorporation of Sr in micropearls, suggest that new micropearls are formed rapidly after cell division. Their formation seems to start in the central zone of the cells and as they grow, they are displaced to the anterior pole of the cells.

Finally, it has been shown that Sr uptake by *T. chui* cells is much higher than that of microalgae that do not form micropearls. *T. chui* cells significantly decreased Sr concentrations in the culture media, supporting the idea of using micropearl‐forming Chlorodendrophyceae species as potential candidates to develop new remediation techniques to treat radioactive ^90^Sr contamination (Møller & Mousseau, 2006; Pathak & Gupta, 2020; Vakulovsky et al., 1994). Alternatively, micropearl‐forming Chlorodendrophyceae could be used to reduce natural Sr levels in drinking water (Agency for Toxic Substances and Disease Registry, 2004; Health Canada, 2019). Ongoing studies will be crucial to assess the suitability of these organisms for these bioremediation purposes.

## CONFLICT OF INTEREST

The authors have no conflict of interest to declare.

## Supporting information


**Appendix S1** Supporting Information

## References

[emi413124-bib-0001] Addadi, L. , Raz, S. & Weiner, S. (2003) Taking advantage of disorder: amorphous calcium carbonate and its roles in biomineralization. Advanced Materials, 15, 959–970.

[emi413124-bib-0002] Agency for Toxic Substances and Disease Registry . (2004) Toxicological profile for strontium. Atlanta, GA: Department of Health and Human Services, Public Health Service. 38527161

[emi413124-bib-0003] Aizenberg, J. , Addadi, L. , Weiner, S. & Lambert, G. (1996) Stabilization of amorphous calcium carbonate by specialized macromolecules in biological and synthetic precipitates. Advanced Materials, 8, 222–226.

[emi413124-bib-0004] Barat Baviera, M. , Ferrús Pérez, M.A. , Font Pérez, G. , Hardisson de la Torre, A. , Herrera Marteache, A. , Marti del Moral, A. et al. (2013) Report of the scientific Committee of the Spanish Agency for food safety and nutrition on a request for initial assessment for marketing of the marine microalgae *Tetraselmis chuii* under regulation (EC) no 258/97 on novel foods and novel food ingredients. Comité Científico de la AESAN, 18, 11–28.

[emi413124-bib-0005] Benzerara, K. , Duprat, E. , Bitard‐Feildel, T. , Caumes, G. , Cassier‐Chauvat, C. , Chauvat, F. et al. (2022) A new gene family diagnostic for intracellular biomineralization of amorphous ca carbonates by cyanobacteria. Genome Biology and Evolution, 14, evac026. 35143662 10.1093/gbe/evac026 PMC8890360

[emi413124-bib-0006] Benzerara, K. , Skouri‐Panet, F. , Li, J. , Férard, C. , Gugger, M. , Laurent, T. et al. (2014) Intracellular ca‐carbonate biomineralization is widespread in cyanobacteria. Proceedings of the National Academy of Sciences of the United States of America, 111, 10933–10938. 25009182 10.1073/pnas.1403510111 PMC4121779

[emi413124-bib-0007] Bidwell, J.P. , Paige, J.A. & Kuzirian, A.M. (1986) Effects of strontium on the embryonic development of *Aplysia californica* . The Biological Bulletin, 170, 75–90. 10.2307/1541824 29314941

[emi413124-bib-0008] Blondeau, M. , Benzerara, K. , Ferard, C. , Guigner, J.‐M. , Poinsot, M. , Coutaud, M. et al. (2018) Impact of the cyanobacterium *Gloeomargarita lithophora* on the geochemical cycles of Sr and Ba. Chemical Geology, 483, 88–97.

[emi413124-bib-0009] Blondeau, M. , Sachse, M. , Boulogne, C. , Gillet, C. , Guigner, J.‐M. , Skouri‐Panet, F. et al. (2018) Amorphous calcium carbonate granules form within an intracellular compartment in calcifying cyanobacteria. Frontiers in Microbiology, 9, 1768. 30127775 10.3389/fmicb.2018.01768 PMC6087745

[emi413124-bib-0010] Brownlee, C. , Langer, G. & Wheeler, G.L. (2020) Coccolithophore calcification: changing paradigms in changing oceans. Acta Biomaterialia, 120, 4–15. 32763469 10.1016/j.actbio.2020.07.050

[emi413124-bib-0011] Cam, N. , Benzerara, K. , Georgelin, T. , Jaber, M. , Lambert, J.F. , Poinsot, M. et al. (2018) Cyanobacterial formation of intracellular ca‐carbonates in undersaturated solutions. Geobiology, 16, 49–61. 29076282 10.1111/gbi.12261

[emi413124-bib-0012] Cam, N. , Benzerara, K. , Georgelin, T. , Jaber, M. , Lambert, J.‐F. , Poinsot, M. et al. (2016) Selective uptake of alkaline earth metals by cyanobacteria forming intracellular carbonates. Environmental Science & Technology, 50, 11654–11662. 27712057 10.1021/acs.est.6b02872

[emi413124-bib-0013] Cam, N. , Georgelin, T. , Jaber, M. , Lambert, J.F. & Benzerara, K. (2015) *In vitro* synthesis of amorphous mg‐, ca‐, Sr‐ and Ba‐carbonates: what do we learn about intracellular calcification by cyanobacteria? Geochimica et Cosmochimica Acta, 161, 36–49.

[emi413124-bib-0014] Couradeau, E. , Benzerara, K. , Gérard, E. , Moreira, D. , Bernard, S. , Brown, G.E. et al. (2012) An early‐branching microbialite cyanobacterium forms intracellular carbonates. Science, 336, 459–462. 22539718 10.1126/science.1216171

[emi413124-bib-0015] De Wever, A. , Benzerara, K. , Coutaud, M. , Caumes, G. , Poinsot, M. , Skouri‐Panet, F. et al. (2019) Evidence of high ca uptake by cyanobacteria forming intracellular CaCO_3_ and impact on their growth. Geobiology, 17, 676–690. 31347755 10.1111/gbi.12358

[emi413124-bib-0016] Decelle, J. & Not, F. (2015) Acantharia. In: eLS. Chichester: John Wiley & Sons, Ltd, pp. 1–10.

[emi413124-bib-0017] Drouet, T. & Herbauts, J. (2008) Evaluation of the mobility and discrimination of ca, Sr and Ba in forest ecosystems: consequence on the use of alkaline‐earth element ratios as tracers of ca. Plant and Soil, 302, 105–124.

[emi413124-bib-0018] Fauré‐Fremiet, E. & Gauchery, M. (1957) Concrétions minérales intracytoplasmiques chez les Ciliés. The Journal of Protozoology, 4, 96–109.

[emi413124-bib-0019] Fuller, W.H. & Hardcastle, J.E. (1967) Relative absorption of strontium and calcium by certain algae. Soil Science Society of America Journal, 31, 772–774.

[emi413124-bib-0020] Gonzalez, M.A. , Aguayo, P.A. , Inostroza, I.D.L. , Castro, P.A. , Fuentes, G.A. & Gomez, P.I. (2015) Ultrastructural and molecular characterization of *Tetraselmis* strains (Chlorodendrophyceae, Chlorophyta) isolated from Chile. Gayana Botanica, 72, 47.

[emi413124-bib-0021] Gray, N.D. & Head, I.M. (2014) The family Chromatiaceae. In: Rosenberg, E. , DeLong, E.F. , Lory, S. , Stackebrandt, E. & Thompson, F. (Eds.) The prokaryotes. Berlin: Springer, pp. 1–14.

[emi413124-bib-0022] Guiry, M.D. & Guiry, G.M. (2021) AlgaeBase [World‐wide electronic publication]. Galway: National University of Ireland.

[emi413124-bib-0023] Hanlon, R.T. , Bidwell, J.P. & Tait, R. (1989) Strontium is required for statolith development and thus normal swimming behaviour of hatchling cephalopods. The Journal of Experimental Biology, 141, 187–195. 2926318 10.1242/jeb.141.1.187

[emi413124-bib-0024] Head, I.M. , Gray, N.D. , Babenzien, H.‐D. & Oliver Glöckner, F. (2000) Uncultured giant sulfur bacteria of the genus *Achromatium* . FEMS Microbiology Ecology, 33, 171–180. 11098068 10.1111/j.1574-6941.2000.tb00739.x

[emi413124-bib-0025] Health Canada . (2019) Guidelines for Canadian Drinking Water Quality: Guideline Technical Document — Strontium. Water and Air Quality Bureau, Healthy Environments and Consumer Safety Branch, Health Canada, Ottawa, Ontario. (Catalogue No ‐ H144‐13/14‐2019E‐PDF).

[emi413124-bib-0026] Jovanović, P. , Rachmilevitch, S. , Roitman, N. & Erel, R. (2021) Strontium as a tracer for calcium: uptake, transport and partitioning within tomato plants. Plant and Soil, 466, 303–316.

[emi413124-bib-0027] Knauss, H. & Porter, J. (1954) The absorption of inorganic ions by *Chlorella pyrenoidosa* . Plant Physiology, 29, 229. 16654647 10.1104/pp.29.3.229 PMC540502

[emi413124-bib-0028] Kylin, A. & Das, G. (1967) Calcium and strontium as micronutrients and morphogenetic factors for *Scenedesmus* . Phycologia, 6, 201–210.

[emi413124-bib-0029] Li, J. , Margaret Oliver, I. , Cam, N. , Boudier, T. , Blondeau, M. , Leroy, E. et al. (2016) Biomineralization patterns of intracellular carbonatogenesis in cyanobacteria: molecular hypotheses. Minerals, 6, 10.

[emi413124-bib-0057] Littler, D.S., Hellebust, J.A., Littler, M.M. & Craigie, J.S. (1973) Handbook of Phycological Methods: Culture methods and growth measurements. In: J.R. Stein (Eds.) New York: Cambridge University Press.

[emi413124-bib-0030] Loste, E. , Wilson, R.M. , Seshadri, R. & Meldrum, F.C. (2003) The role of magnesium in stabilising amorphous calcium carbonate and controlling calcite morphologies. Journal of Crystal Growth, 254, 206–218.

[emi413124-bib-0031] Lowry, R.V. (2015) Website for Statistical Computation . Available at: http://vassarstats.net. [Accessed August 2021].

[emi413124-bib-0032] Luther, G.W., III . (2016) Inorganic chemistry for geochemistry and environmental sciences: fundamentals and applications. New York: John Wiley & Sons.

[emi413124-bib-0033] Mann, S. (2001) Biomineralization: principles and concepts in bioinorganic materials chemistry. New York: Oxford University Press on Demand.

[emi413124-bib-0034] Martignier, A. , De Respinis, S. , Filella, M. , Segovia‐Campos, I. , Marin, B. , Günther, G. et al. (2020) Biomineralization capacities of Chlorodendrophyceae: correlation between chloroplast morphology and the distribution of micropearls in the cell. Protist, 171, 125760. 33126021 10.1016/j.protis.2020.125760

[emi413124-bib-0035] Martignier, A. , Filella, M. , Pollok, K. , Melkonian, M. , Bensimon, M. , Barja, F. et al. (2018) Marine and freshwater micropearls: biomineralization producing strontium‐rich amorphous calcium carbonate inclusions is widespread in the genus *Tetraselmis* (Chlorophyta). Biogeosciences Discussions, 2018, 1–22.

[emi413124-bib-0036] Martignier, A. , Pacton, M. , Filella, M. , Jaquet, J.M. , Barja, F. , Pollok, K. et al. (2017) Intracellular amorphous carbonates uncover a new biomineralization process in eukaryotes. Geobiology, 15, 240–253. 27696636 10.1111/gbi.12213

[emi413124-bib-0037] McFadden, G. & Melkonian, M. (1986) Use of Hepes buffer for microalgal culture media and fixation for electron microscopy. Phycologia, 25, 551–557.

[emi413124-bib-0038] Mei, L. , Xitao, X. , Renhao, X. & Zhili, L. (2006) Effects of strontium‐induced stress on marine microalgae *Platymonas subcordiformis* (Chlorophyta: Volvocales). Journal of Oceanology and Limnology, 24, 154–160.

[emi413124-bib-0039] Meseck, S.L. , Alix, J.H. & Wikfors, G.H. (2005) Photoperiod and light intensity effects on growth and utilization of nutrients by the aquaculture feed microalga, *Tetraselmis chui* (PLY429). Aquaculture, 246, 393–404.

[emi413124-bib-0040] Miledi, R. (1966) Strontium as a substitute for calcium in the process of transmitter release at the neuromuscular junction. Nature, 212, 1233–1234. 21090447 10.1038/2121233a0

[emi413124-bib-0041] Møller, A.P. & Mousseau, T.A. (2006) Biological consequences of Chernobyl: 20 years on. Trends in Ecology & Evolution, 21, 200–207. 16701086 10.1016/j.tree.2006.01.008

[emi413124-bib-0042] Monteil, C.L. , Benzerara, K. , Menguy, N. , Bidaud, C.C. , Michot‐Achdjian, E. , Bolzoni, R. et al. (2020) Intracellular amorphous Ca‐carbonate and magnetite biomineralization by a magnetotactic bacterium affiliated to the Alphaproteobacteria. The ISME Journal, 15, 1–18. 10.1038/s41396-020-00747-3 PMC7853122 32839547

[emi413124-bib-0043] Norris, R.E. , Hori, T. & Chihara, M. (1980) Revision of the genus *Tetraselmis* (Class Prasinophyceae). Shokubutsugaku zasshi, 93, 317.

[emi413124-bib-0044] Pathak, P. & Gupta, D.K. (2020) Strontium contamination in the environment. Cham, Switzerland: Springer.

[emi413124-bib-0045] Riding, R. (2012) A hard life for cyanobacteria. Science, 336, 427–428. 22539710 10.1126/science.1221055

[emi413124-bib-0046] Schneider, C.A. , Rasband, W.S. & Eliceiri, K.W. (2012) NIH image to ImageJ: 25 years of image analysis. Nature Methods, 9, 671–675. 22930834 10.1038/nmeth.2089 PMC5554542

[emi413124-bib-0047] Segovia‐Campos, I. , Martignier, A. , Filella, M. , Jaquet, J.‐M. & Ariztegui, D. (2021) Micropearls and other intracellular inclusions of amorphous calcium carbonate: an unsuspected biomineralization capacity shared by diverse microorganisms. Environmental Microbiology, 24, 537–550. 33817930 10.1111/1462-2920.15498 PMC9292747

[emi413124-bib-0049] Tashmukhamedov, B. , Makhmudova, E. , Bespalko, N. , Kazakov, I. , Polikarpov, G. & Lazarenko, G. (1983) Strontium transport system of brown alga *Cystoseira barbata*: reconstitution on bilayer lipid membranes. General Physiology and Biophysics, 2, 107–110.

[emi413124-bib-0050] Thien, B. , Martignier, A. , Jaquet, J.‐M. & Filella, M. (2017) Linking environmental observations and solid solution thermodynamic modeling: the case of Ba‐and Sr‐rich micropearls in Lake Geneva. Pure and Applied Chemistry, 89, 645–652.

[emi413124-bib-0051] Tibbetts, S.M. , Milley, J.E. & Lall, S.P. (2015) Chemical composition and nutritional properties of freshwater and marine microalgal biomass cultured in photobioreactors. Journal of Applied Phycology, 27, 1109–1119.

[emi413124-bib-0052] Ulloa, G. , Otero, A. , Sánchez, M. , Sineiro, J. , Núñez, M.J. & Fábregas, J. (2012) Effect of Mg, Si, and Sr on growth and antioxidant activity of the marine microalga *Tetraselmis suecica* . Journal of Applied Phycology, 24, 1229–1236.

[emi413124-bib-0053] Vakulovsky, S. , Nikitin, A. , Chumichev, V. , Katrich, I.Y. , Voitsekhovich, O. , Medinets, V. et al. (1994) Cesium‐137 and strontium‐90 contamination of water bodies in the areas affected by releases from the Chernobyl nuclear power plant accident: an overview. Journal of Environmental Radioactivity, 23, 103–122.

[emi413124-bib-0054] Walker, J.B. (1953) Inorganic micronutrient requirements of *Chlorella*: I. Requirements for calcium (or strontium), copper, and molybdenum. Archives of Biochemistry and Biophysics, 46, 1–11. 13092940 10.1016/0003-9861(53)90163-5

[emi413124-bib-0055] Watanabe, T. , Broadley, M.R. , Jansen, S. , White, P.J. , Takada, J. , Satake, K. et al. (2007) Evolutionary control of leaf element composition in plants. The New Phytologist, 174, 516–523. 17447908 10.1111/j.1469-8137.2007.02078.x

[emi413124-bib-0056] Weiner, S. & Dove, P.M. (2003) An overview of biomineralization processes and the problem of the vital effect. Reviews in Mineralogy and Geochemistry, 54, 1–29.

