# Supplementary material for: High calcium and strontium uptake by the green microalga Tetraselmis chui is related to micropearl formation and cell growth
Source: Environ Microbiol Rep. 2022 Sep 23;15(1):38–50. doi: 10.1111/1758-2229.13124 (PMC10103758; doi:10.1111/1758-2229.13124)
Supplement: Supplementary file 1 — Appendix S1 Supporting Information [file EMI4-15-38-s001.docx]

### **Supporting Information**


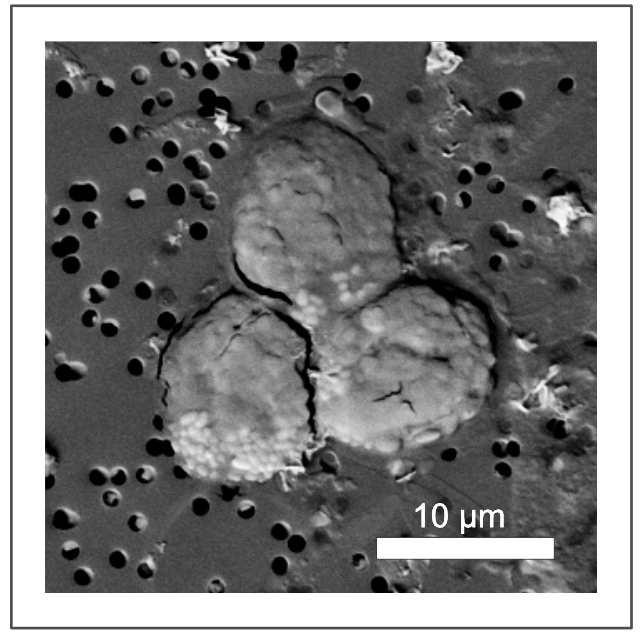


**Figure S1** SEM image of three *T. chui* cells after 288 h of culture in ASP-H medium enhanced with 0.5 mM Ca. Micropearls are clearly visible in the apical pole of cells.

###
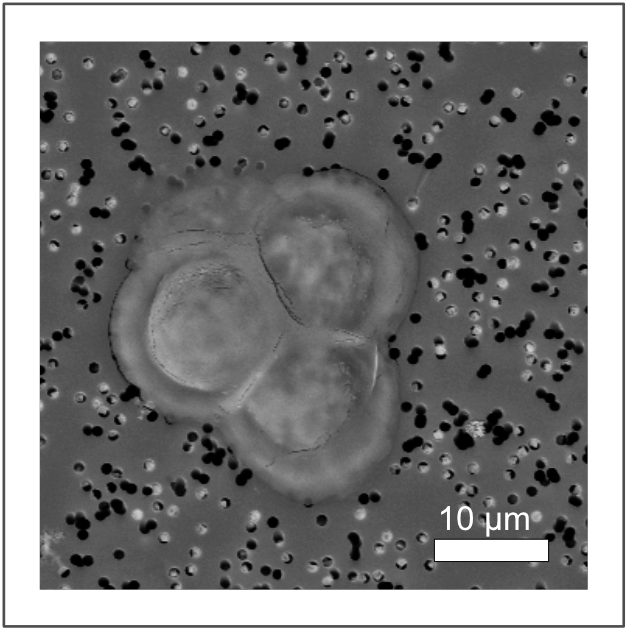


**Figure S2** SEM image of three *T. marina* cells after 288 h of culture in ASP-H medium enhanced with 2.5 mM Ca. Micropearls are not observed.

###
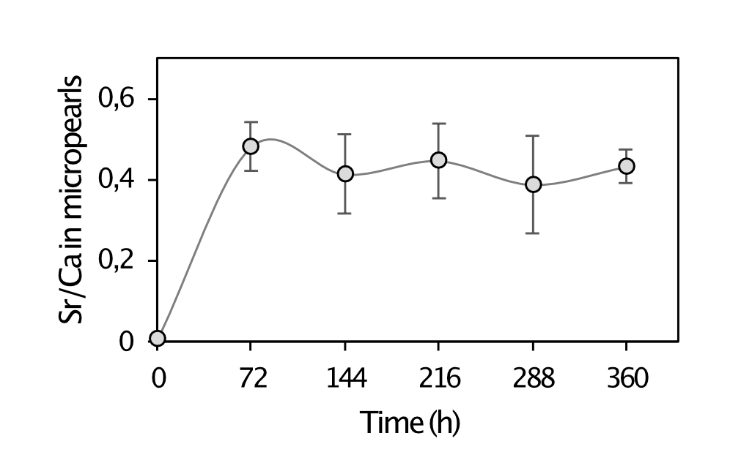


### **Figure S3** Time evolution of Sr/Ca atomic ratios of micropearls contained in T. chui cells cultured in ASP-H medium enhanced with 2.5 mM Ca and 0.1 mM Sr. Each datapoint represents the average value of replicates (the number of replicates varies between 5 and 12). Error bars represent standard deviations between replicates.

**
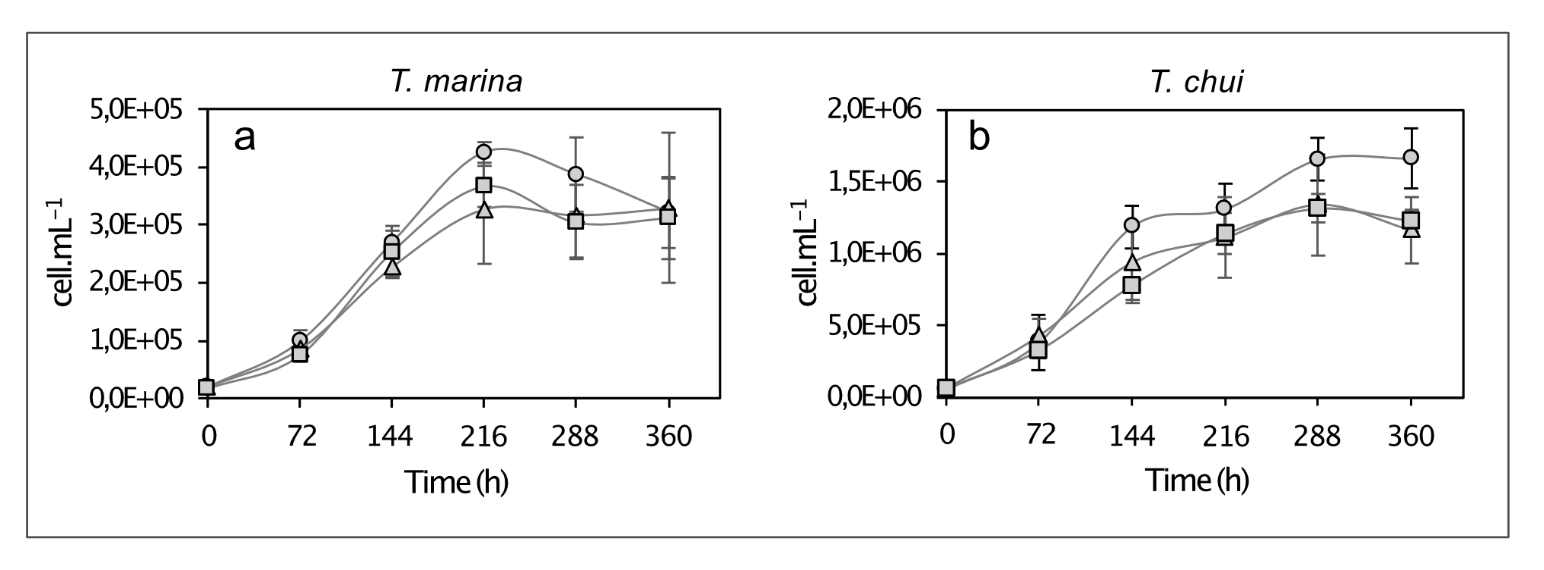
**

**Figure S4** Growth curves of (a) *T. marina* and (b) *T. chui* cultures enhanced with 2.5 mM Ca (squares), 2.5 mM Ca and 0.1 mM Sr (triangles), and 2.5 mM Ca and 0.5 mM Sr (circles). Error bars represent standard deviations (n=3).

###
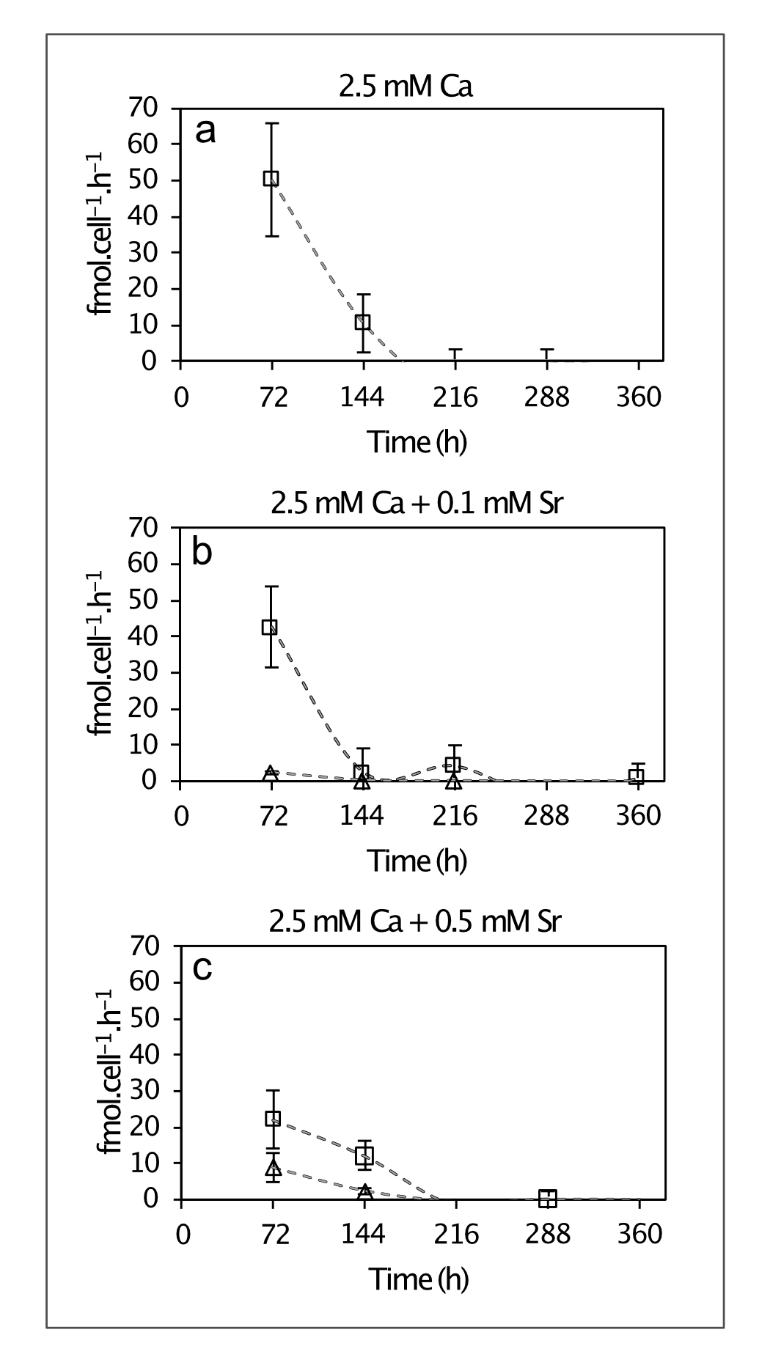


### **Figure S5** Time evolution of T. chui Ca (squares) and Sr (triangles) uptake rates in cultures enhanced with (a) 2.5 mM Ca, (b) 2.5 mM Ca and 0.1 mM Sr, and (c) 2.5 mM Ca and 0.5 mM Sr. Error bars represent standard deviations (n=3).

| Theoretical initial dissolved Sr/Ca (mol%) | 0.04 | 0.2 | 5.0 | 25.0 |
| --- | --- | --- | --- | --- |
| Sr/Ca (mol%) in micropearls after 72 h | 0.42 (±0.1) | 0.46 (±0.2) | 1.07 (±0.2) | 3.77 (±0.8) |
| Sr bioaccumulation factor | 10.5 (±3.0) | 2.3 (±1.0) | 0.21 (±0.04) | 0.15 (±0.03) |

**Table S1** Sr/Ca mol% ratios measured in micropearls (n ≥ 25) of *T. chui* cells cultured between 72 and 288 h in culture media containing different initial dissolved Sr/Ca mol% ratios, and their respective Sr accumulation factor. The Sr bioaccumulation factor was calculated as the ratio between Sr/Ca atomic ratio in micropearls and the initial atomic ratio in the culture medium.

| Species | Strain | Environment | Lifestyle |
| --- | --- | --- | --- |
| *Tetraselmis marina* | CCAC 202.80 | Marine | Non-motile cells forming sessile colonies. |
| *Tetraselmis chui* | CCAC 0014 | Marine | Mainly motile cells presenting four flagella. Non-motile during cell division and unfavorable growth condition. |

### **Table S2** Tetraselmis strains studied in this article.

| ASP-H modified medium without Ca | |
| --- | --- |
| HEPES | 3.3 mM |
| NaCl | 308 mM |
| MgSO_4_⋅ 7 H_2_0 | 20.3 mM |
| KCl | 8.1 mM |
| NaNO_3_ | 0.59 mM |
| K_2_HPO_4_⋅ 3 H_2_O | 22.00 µM |
| Na_2_CO_3_ | 0.3 mM |
| NTA | 52 µM |
| EDTA | 17.86 µM |
| FeSO_4_⋅7 H2O | 17.9 µM |
| KOH | 20 nM |
| ZnCl_2_ | 2.4 µM |
| MnCl_2_⋅ 4 H_2_O | 21.9 µM |
| CoCl_2_⋅ 6 H_2_O | 0.51 µM |
| Na/EDTA | 69.3 µM |
| H_3_BO_3_ | 0.48 mM |
| Vitamine B12 | 0.15 nM |
| Biotine | 4.1 nM |
| Thiamine-HCl | 0.3 µM |
| Niacinamide | 0.8 nM |

### **Table S3** Chemical composition of the ASP-H medium (Stein, 1979) modified by McFadden and Melkonian (1986) without CaCl_2_.
